# Evaluation of chronic pruritus and associated skin findings in patients with diabetes mellitus

**DOI:** 10.55730/1300-0144.5716

**Published:** 2023-09-09

**Authors:** Fatma AVCI MERDİN, Ahmet Taha AYDEMİR, Fatma Nur KORKMAZ, Fatma Tuğçe ŞAH ÜNAL, Asena GÖKÇAY CANPOLAT, Ayşe ÖKTEM, Özgür DEMİR, Mustafa ŞAHİN, Rıfat EMRAL, Murat Faik ERDOĞAN, Sevim GÜLLÜ, Vedia TONYUKUK GEDİK, Pelin KOÇYİĞİT, Demet ÇORAPÇIOĞLU

**Affiliations:** 1 Division of Endocrinology and Metabolism, Department of Internal Medicine, Faculty of Medicine, Ankara University, Ankara, Turkiye; 2 Department of Dermatology and Venereal Diseases, Faculty of Medicine, Ankara University, Ankara, Turkiye

**Keywords:** Bullous pemphigoid, diabetes mellitus, pruritus

## Abstract

**Background/aim:**

To explore the dermatological lesions associated with chronic pruritus in patients who were followed up at our clinic for type 1 and type 2 diabetes mellitus (DM).

**Materials and methods:**

The study population consisted of 249 patients with DM, who presented to the endocrinology clinic at Ankara University Faculty of Medicine between January 2022, and March 2022, regardless of whether they had reported experiencing pruritus symptoms. The visual analog scale and 5-D itch scale were used to determine the severity of itching in patients. Dermatological examination findings were also evaluated.

**Results:**

Of the 249 patients with DM, mean duration since diabetes was diagnosed was 12 ± 9.2 [median 10 (0.3–46)] years, and the mean HbA1c levels were 8.1% ± 2.1%. Pruritus was detected in 77 (30.9%) patients and the mean duration of diabetes diagnosis was 13.4 ± 9.7 years. Examination of the microvascular and macrovascular complications showed that the incidence of retinopathy, nephropathy, neuropathy and peripheral arterial disease was 31.2% (p = 0.003), 31.2% (p = 0.005), 66.2% (p < 0.001) and 10.4% (p = 0.038), respectively, in the group with pruritus. These incidences were significantly higher in the group with pruritus than in those without pruritus. Dermatological examination showed that the most common condition was xerosis (64%), followed by fungal skin infection (16%) and bullous pemphigoid (8%). No skin findings were noted in 7% of patients who complained of itching.

**Conclusion:**

Chronic pruritus may be associated with several factors such as poor glycemic control, high BMI and microvascular and macrovascular complications in patients with DM. Especially in patients with severe generalized pruritus who do not respond to standard antipruritic treatments, the use of DPP-4 inhibitors, a class of oral antidiabetic agents, should be questioned and all medications being used by the patient should be reviewed.

## 1. Introduction

Diabetes mellitus (DM) is a chronic metabolic disease characterised by hyperglycemia, which occurs due to impaired insulin secretion, insulin action, or both. In patients with diabetes mellitus, complications affecting all organs, especially the vascular system, may occur in relation with the degree and duration of hyperglycemia during the disease process. Several skin manifestations may occur with etiology and mechanisms comparable to the complications that develop in patients with DM. While skin lesions such as diabetic dermopathy, necrobiosis lipoidica diabeticorum are only or often seen in patients with DM, symptoms such as generalized itching, palmar erythema, and facial flushing are nonspecific skin disorders that may accompany diabetes [1–3]. Chronic itching is usually the result of microcirculation problems and/or insufficient sweating [2]. Furthermore, concomitant autoimmune diseases, reactions to medications, and opportunistic infections may also cause serious itching [2]. While the incidence of chronic pruritus is 16%–23% in the general population, it is estimated to range between 3% and 49% in patients with DM [4–6]. Pruritus, or itching, could be a chronic and debilitating condition for people with diabetes. It can disrupt sleep, cause fatigue, lead to social isolation, and make it difficult to focus [7]. Chronic pruritus remains an important problem that might adversely affect the quality of life in patients with diabetes. Therefore, a better understanding of the risk factors of pruritus in patients with diabetes may increase awareness.

We examined demographic characteristics of patients followed at our clinic for type 1 and type 2 DM suffering from chronic itching and the presence of other factors that may cause chronic itching, as well as evaluating their skin findings aiming to raise awareness over chronic itching, which is a commonly overlooked issue.

## 2. Materials and methods

The study population consisted of 249 patients, aged 18 years and above, who were diagnosed with either type 1 or type 2 DM. This included individuals who presented to the endocrinology clinic at Ankara University Faculty of Medicine between January 2022, and March 2022, regardless of whether they had reported experiencing pruritus symptoms. This was a cross-sectional study. Patients who were under the age of 18 or did not have a diagnosis of diabetes mellitus were excluded from the study sample.

In order to determine the severity of pruritus in patients who reported experiencing itching, two scales were used: the visual analogue scale (VAS) and the 5-D itch scale, which have been validated for use in the Turkish population [8–10]. The VAS is a 10-cm line that can be oriented horizontally or vertically for a subjective assessment of pruritus [8]. Patients were instructed to mark the line at the point that represented the intensity of their pruritus, with the beginning of the scale indicating no pruritus (0 points) and the end indicating the most severe pruritus they could imagine (10 points) [8]. Using a numerical rating scale, patients verbally assessed the intensity of their itching on a scale from 0 (no itching) to 10 (the worst itch imaginable) [8]. Additionally, patients with itching were evaluated by the dermatology doctors at Ankara University Faculty of Medicine (A.T.A, A.Ö, P.K ) and the dermatological findings were recorded.

The study was approved by Ankara University, School of Medicine, Non-Invasive Clinical Research Ethics Committee (statement number: 2021/462) and informed consent was obtained from all patients.

## 3. Statistical analysis

All data analyses were carried out using the Statistical Package for Social Sciences (IBM Corp. 2017. Version 25.0. Armonk, NY, USA). The conformity of the variables to the normal distribution was examined through visual (histogram and probability graphs) and analytical methods (Kolmogorov-Smirnov/Shapiro-Wilk tests). Descriptive analyses were presented in mean and standard deviation values for normally distributed numerical variables, median values, extreme values, and interquartile range for nonnormally distributed numerical variables, and frequency tables for ordinal and categorical variables. In comparisons between groups, the Mann-Whitney U test was used for numerical variables that were not normally distributed, and Chi-square or Fisher Tests were used for categorical variables, whichever was appropriate. A multivariate analysis was performed to identify the factors that independently contribute to the development of pruritus. Logistic regression was employed to examine the association between the independent variables and pruritus development. This analysis included parameters that were found to be statistically significant or approaching significance (p ≤ 0.10) in the initial univariate analysis. The model fit was evaluated through the Hosmer-Lemeshow test. Statistical significance was determined at a threshold of p < 0.05, indicating that results with a p-value below this threshold were considered statistically significant.

## 4. Results

Of the 249 patients, 228 had Type 2 DM and 21 had Type 1 DM. There were 155 females and 94 males, and the mean time duration since diabetes diagnosis was 12 ± 9.2 years, and the mean HbA1c levels were 8.1% ± 2.1%. Patients with no itching complaints were categorized as Group 1, and patients reporting itching were named as Group 2. The itching was detected in 77 (30.9%) of the patients included in the study. Of these patients, 48 were female and 29 were male, and the mean duration since diabetes diagnosis was 13.4 ± 9.7 years. The mean BMI was measured as 31.3 ± 5.8 kg/m2 (p = 0.011) in the patient group with chronic itching, and it was found to be significantly higher than in the group with no itching (Table 1).

Examination for the microvascular and macrovascular complications in both groups revealed that the incidence of retinopathy, nephropathy, neuropathy, and peripheral arterial disease was 31.2% (p = 0.003), 31.2% (p = 0.005), 66.2% (p < 0.001) and 10.4% (p = 0.038), respectively in Group 2. These complications were significantly higher in Group 2 than in Group 1. When the antidiabetic treatments given in both groups were examined, the incidence of itching was significantly higher in the group receiving mixed insulin or basal-bolus insulin treatment with 46.5% (p: 0.032) (Table 2).

In our evaluation of the severity of itching with the visual analog scale, the median value was found to be 7 in our patients. In the survey that we conducted with the 5-D itching scale, 70% of the patients with itching reported itching that lasted less than 6 ha day. Besides, 36% of the patients with itching reported itching persisted all day despite various treatments (Table 3). Widespread pruritus over the entire body occurred in 27.2% of patients with itching. The most common body parts where itching was experienced were the lower legs (15%) and points of pressure with clothing (9%) (Table 3).

Dermatological evaluations of the patients with chronic pruritus revealed that 64% had xerosis, 16% fungal skin infection, 8% bullous pemphigoid, 5% stasis dermatitis, 3% acute contact dermatitis, 1% neurodermatitis, %1 prurigo nodularis, 1% chronic urticaria, and 1% diabetic dermopathy (Table 4). The most common cutaneous finding in patients with pruritus was xerosis cutis with a rate of 64%. No skin findings were noted in 7% of patients who complained of itching. When we evaluated pruritus severity using the visual analog scale, 18.1% (14) of patients with itching had a severity of 10. The median age of patients with severe pruritus was 74 (69–85) years, and all patients with severe pruritus had type 2 DM. Of the patients with severe pruritus, 59% had xerosis, 18% had fungal dermatitis, 18% had bullous pemphigoid, 6% had acute contact dermatitis and 6% had chronic dermatitis. In addition, sleep delay due to itching was observed in 28% of the patients with severe pruritus.

In our study, logistic regression analysis was applied to determine the independent risk factors for the development of pruritus. We found no statistically significant effect of HbA1c, BMI, type of insulin treatment, or concurrent diabetic complications on skin findings in patients with itching. In the presence of distal symmetric sensory polyneuropathy [OR (95% CI): 2.321 (1.152–4,672), p: 0.018], high serum anti-thyroglobulin (anti-TG) [OR (95% CI): 2.578 (1.009–6.585), p: 0.002], and elevated gamma glutamyl transferase (GGT) enzyme [OR (95% CI): 3.478 (1.592–7.594), p: 0.048] were independent risk factors for the development of pruritus in patients with diabetes mellitus in our study.

## 5. Discussion

Itching that lasts longer than six weeks is defined as chronic pruritus, and it affects the quality of life in patients. Although the risk of itching in the generalized form increases in diabetic patients, it has been detected in the localized form of itching, and itching localizing in the torso with no known reason has been reported to be more common in diabetic patients [11,12]. Neilly et al. investigated the presence of generalized and localized pruritus by comparing 300 DM patients of similar age and gender distribution with 100 nondiabetic healthy controls [13]. Although generalized pruritus was detected in 8 of the diabetic patients (2.7%), the presence of localized pruritus was similar in both groups [13]. In our study, we evaluated 249 patients with DM and detected itching problems in 77 (30.9%) of them. Fourteen of our patients who reported the complaint of itching were in the generalized form, and when evaluated in detail, we found that the other patients had itching in the localized form, with the most common body parts being the lower legs at 15% and points of contact with clothing at 9%. Meijuko et al. evaluated the characteristics and intensity of pruritus in 385 patients with type 2 DM using a detailed interview questionnaire including a visual analog scale [14]. They found generalized itching in 27.5% of the patients, and 24.5% of these patients had difficulty in falling asleep due to itching, while 15.1% reported that they had sleep disorders [14]. When they questioned the severity of itching with the visual analog scale (VAS), researchers observed that itching in the generalized form had a higher effect on sleep and mood in patients with a higher VAS score [14]. In our study, the median score was calculated as 7 based on the data from the questions about the severity of itching through the visual analog scale. When we evaluated pruritus severity using a visual analog scale, 18.1% (14) of patients had a severity of 10. The median age of patients with severe pruritus was 74 (69–85) years, and all patients with severe pruritus had type 2 DM. Consistent with the findings in the literature, 28% of patients with severe pruritus in our study experienced delayed sleep onset due to pruritus, while 18% experienced sleep disturbances. Skin findings are common in diabetic patients, and skin problems have been reported in approximately 30% to 79% of patients [15–17]. A study conducted with 750 diabetic patients, for instance, found that the most common skin findings were cutaneous infections (47.5%), xerosis (26.4%), and inflammatory skin diseases (20.7%) [16]. In a study conducted by Aktaş et al, xerosis (40.52%) was found as the most common skin finding in 153 patients with type 2 DM, and other common skin findings included tinea pedis (18.95%), onychomycosis (9.80%), pruritus (14.38%) and seborrheic keratosis (10.46%) [18]. Likewise, Goyal et al examined 200 diabetic patients, reporting that the most common issues included xerosis (44%), diabetic dermopathy (36%), skin tags (32%), cutaneous infections (31%), and seborrheic keratosis (30%) [19]. Examination of the skin findings for our patients with itching showed that the most frequent symptom was xerosis at 64%, followed by fungal skin infection at 16%, bullous pemphigoid at 8%, and stasis dermatitis at 5%. Xerosis cutis, also known as abnormal dryness of the skin and often seen in older individuals, can be caused by an underlying secondary disease, dietary factors, and certain medications. Xerosis cutis is characterized by a failure of the stratum corneum (SC) to maintain an adequate gradient of water concentration between the viable epidermal cells and the surface of the skin [20]. In patients with diabetes, the skin’s cellular biochemistry, including microvascular blood flow, sweat production, collagen structure, and function, may be impaired. In our study, we found that the rate of microvascular complications and peripheral artery disease was higher in patients with pruritus, but there was no statistically significant relationship between the presence of xerosis and microvascular complications. Other research has suggested that people with poorly controlled diabetes and accompanying microvascular and macrovascular complications may have a higher incidence of superficial fungal infections such as onychomycosis and tinea pedis [21,22]. However, a study by Romano et al. found no correlation between dermatophyte infections and diabetes duration, HbA1c levels, or glucose levels in 171 diabetic patients, thus contradicting the previously mentioned suggestion that these infections may be more common in people with poorly controlled diabetes [23]. Similarly, the mean HbA1c level of patients with itching in our study was 8.4 ± 2.4, while 15% of them had tinea pedis and onychomycosis. However, there was no significant relationship between HbA1c levels, accompanying microvascular and macrovascular complications, and the presence of tinea pedis and onychomycosis. People with diabetes are at an increased risk for mycobacterial infections due to disruptions in the cellular immune system. A study conducted by Bacakoğlu et al. found that the incidence of diabetes mellitus and tuberculosis was around 12.3% in Turkey [24]. In our study, on the other hand, one patient with pruritus was diagnosed with tuberculosis following further evaluation. Age, duration of diabetes, poor glycemic control, and accompanying complications are known risk factors for the development of tuberculosis in patients with diabetes.

In addition, the reactions related to the drugs used by the patients could also be one of the causes of itching, and in this respect, the patients should be examined in detail. Drug-related reactions may manifest as itching without accompanying skin lesions, as well as drug reactions characterized by skin lesions (cutaneous drug side effects, drug eruptions). Itching may occur due to sulfonylureas, metformin, DPP-4 inhibitors, and insulin drugs used in diabetic patients. On top of that, certain drug classes such as antihypertensives (ACE inhibitors, ARBs, beta blockers) and statins, frequently used in diabetic patients due to comorbidities, are also known to cause itching. We detected bullous pemphigoid in 8% of patients who experienced pruritus and were treated with DPP-4 inhibitors, and it was observed that the patients’ symptoms regressed after changing treatment. Bullous pemphigoid is an autoimmune subepithelial disease characterized by pruritus followed by urticarial plaques and finally bullae on the skin and mucosa. Bullous pemphigoid particularly affects elderly individuals. A meta-analysis conducted in 2018 reported that the incidence of bullous pemphigoid was 3 times higher in patients with type 2 DM using DPP-4 inhibitors [25]. The surge in the incidence of bullous pemphigoid (BP) over recent years has been attributed to overall growth in the elderly population and to the increased use of medications that have the potential to induce BP in patients in this population segment. A case-control study by Kridin et al. has demonstrated an elevated risk of bullous pemphigoid in patients with diabetes mellitus (DM) following the use of vildagliptin and, to a lesser extent, linagliptin [25]. Such association with the gliptins was shown to be independent of the exposure to metformin. However, some studies have suggested that the combination of metformin and dipeptidyl peptidase 4 (DPP-4) inhibitors could induce bullous pemphigoid [26,27]. The time for the development of BP lesions after medication ranged from 10 days to 2 years [26]. In our study, all 6 patients with BP were male. The mean age was 71.8 years, the mean duration of diabetes diagnosis was 15.3 years, and the mean HbA1c level was 8.6%. Three patients with diabetes mellitus received linagliptin + insulin glargine, two patients were treated with vildagliptin + metformin, and one patient was treated with metformin + sitagliptin. In agreement with previous findings in the literature, bullous pemphigoid was detected in patients approximately 13.1 (between 6 and 24) months after the onset of their complaints of pruritus. Although the clinical manifestation of bullous pemphigoid can be highly variable and complex, this immunobullous skin disorder typically presents with tense bullae and intense generalized pruritus. In this study, 18% of the patients with severe pruritus were diagnosed with bullous pemphigoid. Typical bullous lesions appeared with persistent pruritus in 5 of the patients, and the diagnosis was confirmed in one patient by biopsy obtained from prurigo nodularis-like lesions. Lesions and complaints of pruritus regressed in all patients after a change in treatment. Particularly in the elderly population, the mortality rate of bullous pemphigoid may rise by approximately 10% to 40% [28]. For this reason, BP should be suspected in diabetic patients receiving DPP-4 inhibitors for severe pruritus, and medication intake should be investigated in greater detail. Insulin therapy is prescribed in patients who cannot achieve glycemic control with oral antidiabetic therapy, and it is well established that these cases are often accompanied by micro-macrovascular complications. In our study, the complaint of pruritus was significantly higher in the mixed insulin or basal-bolus insulin treatment group at a rate of 46.5% (p = 0.032). However, there was no statistically significant difference between the groups with and without pruritic complaints in terms of the time of diabetes diagnosis, fasting blood sugar, postprandial glucose, and HbA1c levels. A deeper look into insulin preparations used to provide glycemic control suggests that the presence of bovine or pork proteins, the insulin molecule itself, preservatives, or additives may cause allergic reactions [29]. However, although the emergence of recombinant insulin preparations has largely eliminated the-once-common insulin allergies [30], the results of our study showed that more attention should be paid to this issue. Especially in patients with severe generalized pruritus who do not respond to standard antipruritic treatments, the use of DPP-4 inhibitors, one class of oral antidiabetic agents, should be questioned and all medications used by the patient should be reviewed. In addition, a diagnostic biopsy of the skin lesions should be performed, if necessary, and then the specimens should be evaluated by immunofluorescence microscopy.

Despite a high incidence of itching in patients with DM, there is still insufficient evidence of the relationship between glycemic control and itching. As for the pathogenesis, it is generally thought that peripheral neuropathy due to microcirculation disorder and decreased sweating cause chronic itching in diabetic diseases [2,14]. Particularly, peripheral neuropathy is one of the most common microvascular complications in patients with type 1 and type 2 DM. In the examination of microvascular and macrovascular complications in our study, the incidence of retinopathy was 31.2% (p: 0.003), nephropathy 31.2% (p: 0.005), neuropathy 66.2% (p < 0.001), and peripheral artery disease 10.4% (p: 0.038) which were significantly higher in the group with itching complaints. A study conducted by Yosipovitch et al. with 238 patients with type 1 DM found that the duration of diabetes was effective in the development of skin symptoms, and it was associated with the development of diabetic microvascular complications, a finding appears to agree with our results [31]. However, in our study, there was no significant difference between patients with pruritus and patients who had no pruritus complaint in terms of fasting blood glucose levels, postprandial blood glucose levels, and hba1c levels. However, in contradiction with our findings, a study conducted by Meijuko et al. reported that the incidence of itching was significantly higher in patients with type 2 DM who had higher postprandial glucose levels rather than fasting blood glucose and hba1c levels [14]. It has been suggested here that postprandial glucose levels may be a good predictive factor for itching in the generalized form [14]. In concordance with previous work in the literature, our study found evidence that macrovascular and microvascular complications are associated with itching in diabetic patients. It is well established that as the body mass index increases so does insulin resistance, and the regulation of hyperglycemia is impaired in diabetic patients. In our study, the mean BMI was measured as 31.3 ± 5.8 (p: 0.011) in the patient group with chronic itching, and it was found to be significantly higher than the group with no itching. Thus, in addition to various medical treatments for the elimination of itching, patients should be encouraged to lose some weight, which may afford provide better blood sugar control.

The limitations of our study include the fact that the study was conducted in a single center and had a relatively small sample size.

In conclusion, a number of skin symptoms, particularly pruritus, may occur in diabetic patients the etiology and mechanism of these symptoms may be similar to the complications of diabetes. In this context, it is particularly important to keep in mind that chronic pruritus may be associated with a number of factors, such as poor glycemic control, a high BMI, the presence of microvascular and macrovascular complications in patients with DM, as well as the involvement of other dermatological diseases and various systemic complications associated with DM. Additionally, the intake of medications should be investigated in a thorough manner, since particularly elderly patients on DPP-4 inhibitors may be at risk for the development of bullous pemphigoid, and therefore these patients ought to be informed and educated about this potential risk.

## Figures and Tables

**Table 1:** Demographic characteristics and clinical findings of the patients.

	All patients n = 249 (%)	Group 1 n = 172 (%)	Group 2 n = 77 (%)	P-value
Sex*				0.99^a^
Female	155 (62.2)	107 (62.2)	48 (62.3)	
Male	94 (37.8)	65 (37.8)	29 (37.7)	
Age				0.021^b^
Mean ± SD	56.3 ± 13,1	55.3 ± 12.6	57.7 ± 14	
Median (min-max)	58 (19–86)	56.5 (19–82)	61 (20–86)	
DM type*				0.46^a^
Type 1 Diabetes Mellitus	21 (8.4)	13 (7.6)	8 (10,4)	
Type 2 Diabetes Mellitus	228 (91.6)	159 (92.4)	69 (89.6)	
Time since diagnosis (years)				0.13^b^
Mean ± SD	12 ± 9.2	11.3±9	13.4 ± 9.7	
Median (min-max)	10 (0.3–46)	10 (0.3–46)	10 (0.3–40)	
Duration of itching (month)				
Mean ± SD			25.2 ± 36.1	
Median (min-max)			12 (1–180)	
Body mass index (kg/m2				0.011^b^
Mean ± SD	29.9 ± 5.9	29.3 ± 5.8	31.3 ± 5.8	
Median (min-max)	30(17.3–53)	29.2(17.3–53)	31.6 (21.3–49.4)	
Systolic blood pressure, mmHg				0.75^b^
Mean ± SD	125 ± 13.6	125 ± 13.7	124.8 ± 13.4	
Diastolic blood pressure, mmHg				0.32^b^
Mean ± SD	75.2 ± 8.5	75.5 ± 8.4	74.5 ± 8.8	
Smoking history*	76 (30.5)	51 (29.7)	25 (35.2)	0.58^a^
Cigarettes, pack/year				0.62^b^
Mean ± SD	25.1 ± 15.7	25 ± 13.6	25.3 ± 19.4	
Alcohol consumption*	16 (6.4)	8 (4.7)	8 (10.4)	0.099^c^
Daily water consumption (lt)				0.11^b^
Mean ± SD	2.1 ± 0.9	2 ± 0.8	2.2 ± 1.1	
Fasting glucose, mg/dl				0.50^b^
Mean ± SD	151.2 ±59.4	147.6 ± 53.6	159,5 ± 70.4	
Postprandial glucose, mg/dl				0.95^b^
Mean ± SD	218.3 ±74.7	217.1 ± 69.1	221.1 ± 86.4	
HbA1c %				0.34^b^
Mean ± SD	8.1 ± 2.1	8 ± 2	8.4 ± 2.4	
Median (min-max)	7.5(5.4–9.1)	7.2 (5.7–17.7)	7.9 (5.4–19.1)	

n: Number of patients,

* Continuous variables presented as ‘mean ± standard deviation (minimum-maximum)’ and categorical variables presented as ‘number (percent of column),’

a Pearson chi-square Test;

b Mann–Whitney U test;

c Fisher’s exact test.

P < 0.05 was considered statistically significant.

**Table 2:** Comparison of the clinical findings of patients in Groups 1 and 2.

	All patients n = 249(%)	Group 1 n = 172(%)	Group 2 n = 77(%)	P-value
**Comorbidities** *				
Hypertension	164 (65.9)	111 (64.5)	53 (68.8)	0.51^a^
Hyperlipidemia	88 (35.3)	59 (34.3)	29(37.7)	0.61^a^
Thyroid diseases	50 (20.1)	36 (20.9)	14 (18.2)	0.62^a^
Hepatosteatosis	17 (6.8)	14 (8.1)	3 (3.9)	0.22^c^
Autoimmune diseases and allergies	52 (20.9)	32 (18.6)	20 (26)	0.19^a^
**Diabetes treatment** *				
Oral diabetes medications (metformin, gliclazide, DPP-4 inhibitors, SGLT-2 inhibitors)	215 (86.3)	150 (87.2)	65 (84.4)	0.55^a^
DPP4 (sitagliptin, vildagliptin, linagliptin)	107 (43)	73 (42.4)	34 (44.2)	0.80^a^
Basal insulin only (insulin glargine, insulin detemir)	27 (10.8)	19 (11)	8 (10.4)	0.88^a^
Mixed/basal-bolus insulin (insulin aspart protamine, insulin aspart, insulin degludec, insulin lispro, insulin, lispro protamine, insulin glulisine)	88 (35.3)	55 (32)	33 (46,5)	0.032^a^
Antihypertensive drug use*	144 (57.8)	94 (54.7)	50 (64.9)	0.129^a^
Antihyperlipidemic drug use*	88 (35.3)	56 (32.6)	32 (41.6)	0.17^a^
**DM complications** *				
Retinopathy	50 (20.1)	26 (15.1)	24 (31.2)	0.003^a^
Nephropathy	51 (20,5)	27 (15.7)	24 (31.2)	0.005^a^
Neuropathy	112 (45)	61 (35.5)	51 (66.2)	<0.001^a^
Arteriosclerotic heart disease	47 (18.9)	30 (17.4)	17 (22.1)	0.39^a^
Peripheral arterial disease	14 (5.6)	6 (3.5)	8 (10.4)	0.038^c^
Cerebrovascular disease	6 (2.4)	4 (2.3)	2 (2.6)	>0.99^c^

n: Number of patients,

* Continuous variables presented as ‘mean ± standard deviation (minimum-maximum)’ and categorical variables as ‘number (percent of column)’,

a Pearson Chi-Square Test;

b Mann–Whitney U test;

c Fisher’s Exact Test.

The results were considered statistically significant for p < 0.05. DPP-4; Dipeptidyl peptidase-4 enzyme, SGLT-2: sodium glucose cotransporter type 2.

**Table 3:** Data from the 5-D itch scale.

5-D Pruritus Scale		N (77)	%

1. Duration	1 Less than 6 h/day	54	70
	2 6–12 h/day	10	13
	3 12–18 h/day	3	4
	4 18–23 h/day	2	3
	5 All day	8	10

2. Degree	1 Not present	4	5
	2 Mild	23	30
	3 Moderate	24	31
	4 Severe	21	27
	5 Unbearable	5	7

3. Direction	1 Completely resolved	7	9
	2 Much better, but still present	18	24
	3 Slightly better, but still present	17	22
	4 Unchanged	28	36
	5 Getting worse	7	9

4. Disability	1 Never affects this activity	30	39
	2 Rarely affects this activity	23	30
	3 Occasionally affects this activity	5	6
	4 Frequently affects this activity	13	17
	5 Always affects this activity	6	8

5. Distribution	1 Head/Scalp	18	4
	2 Face	18	4
	3 Chest	23	5
	4 Abdomen	33	8
	5 Back	33	8
	6 Buttocks	22	5
	7 Thighs	21	5
	8 Lower legs	63	15
	9 Soles	20	5
	10 Palms	18	4
	11 Tops of Hands/Fingers	35	8
	12 Forearms	32	8
	13 Upper Arms	32	8
	14 Points of pressure under the clothing (e.g., waistband, undergarment) and groin	36	9
	15 Tops of the Feet/Toes	15	4

n: Number of patients

**Table 4:** Distribution of dermatological findings in patients with chronic pruritus.

Dermatological findings	Number of patients (%)
Xerosis	51 (64)
Fungal skin infection	13 (16)
Bullous pemphigoid	6 (8)
Stasis dermatitis	4 (5)
Acute contact dermatitis	2 (3)
Prurigo nodularis	1 (1)
Chronic urticaria	1 (1)
Diabetic dermopathy	1 (1)
Neurodermatitis	1(1)
